# Bibliographical

**Published:** 1895-01

**Authors:** 


### EDITORIAL.

### Bibliographical.

The Anatomy and Pathology of the Teeth, by C. F. W. Bodecker, D. D. S., M. D. S., with three hundred and twenty-five illustrations. Adopted by the National Association of Dental Faculties as a text-book for dental students. From the press of The S. S. White Dental Manufacturing Company, Philadelphia.

The preparation of this work has occupied a large share of the spare time of the author during the past eighteen years. About ten years have been industriously given to the preparation of the matter which composes the volume.

The line of work embraced in the volume is one for which Dr. Bodecker has always entertained a very great love, and in which he has engaged with great earnestness and industry; and in addition to this he has made available the attainments of men eminent in the same line, such as: Dr. Carl Heitzmann, Dr. Wm. H. Atkinson, Dr. Frank Abbott and others.

The work consists of forty-eight chapters, embracing the anatomy of the teeth and adjacent parts, their histology, growth and development, also the abnormal and diseased conditions to which the teeth and surrounding parts are subject.

The work is prepared with special reference to the needs of the dental student, and will be an invaluable addition to his resources in his study and investigation on these various subjects. This work is a valuable one for the practitioner as well as the student, and should be in the library of every dentist.

To Dr. Bodecker the profession is under an obligation in this matter, which they can in part at least repay by conscientious study and thorough understanding of all the subjects that are embraced in the work.

The work is obtainable through any bookseller, or of any of the dental depots. Price $5.00.

